# Health condition and familial factors associated with health-related quality of life in adolescents with congenital heart disease: a cross sectional study

**DOI:** 10.1186/s12955-018-0841-y

**Published:** 2018-01-10

**Authors:** Yu-Mi Im, Tae-Jin Yun, Sunhee Lee

**Affiliations:** 1 Seoul Women’s College of Nursing Seoul, 38, Ganhodae-ro, Seodaemun-gu, Seoul, 03617 South Korea; 2 Division of Pediatric Cardiac Surgery, University of Ulsan College of Medicine, Asan Medical Center, 88, Olympic-ro 43-gil, Songpa-gu, Seoul, 05505 South Korea; 3 College of Nursing, the Catholic University of Korea, 222 Banpo-daero, Seocho-gu, Seoul, 06591 South Korea

**Keywords:** Siblings, Quality of life, Adolescent, Cardiovascular abnormalities

## Abstract

**Background:**

The focus of clinical care after the repair of congenital heart disease has shifted from saving life of the patient to the patient’s quality of life. The purpose of this study was to examine the health condition and familial factors associated with the health related quality of life of adolescents with congenital heart disease.

**Methods:**

Ninety-eight adolescents aged 13–19 years were collected from a congenital heart clinic from July 22 to August 23, 2013. Perceptions of parental rearing behaviors, health related quality of life of adolescent with congenital heart disease, and general characteristics were measured. We used multiple linear regression analysis to explore factors that are associated with the health related quality of life of adolescents with congenital heart disease.

**Results:**

New York heart association class (Adj R^2^ = .186, *p* = .000), presence of siblings (Adj R^2^ = .240, *p* = .010), and mother’s emotional warmth (Adj R^2^ = .265, *p* = .043) were significantly associated with the health related quality of life of adolescents with congenital heart disease.

**Conclusions:**

Emotionally warm parental rearing behaviors and the presence of siblings were important familial factors that were positively associated with HRQOL in adolescents with CHD. Therefore, it is important for healthcare providers to develop a greater sensitivity to, and awareness of, the familial influences that may be impacting a subject’s HRQOL, as well as the exigencies of the CHD, itself.

## Background

Advances in medical and surgical management have improved the life expectancy of children with congenital heart disease (CHD), and have resulted in a significant reduction in mortality [1]. More than 90% of CHD patients survive into adulthood in Korea as well as in US [2, 3]. Due to these increased survival rates, the focus of clinical care after the repair of CHD has shifted from saving the patient’s life to the patient’s quality of life (QOL). Despite improved life expectancy, uncertainty about the course of illness and prognosis, signs and symptoms of the illness, and restrictions in activity levels nevertheless intrude on patient daily life [4–6]. Some patients also struggle with psychological, educational, or behavioral issues [7]. In addition, many children and adolescents live with morbidities associated with an underlying cardiac lesion and/or its treatment, and such morbidities may impact the health-related QOL (HRQOL) of CHD patients [1]. To date, a lot of studies of the QOL in patients with CHD were conducted, but the results have been inconsistent [8]. Yoon and Lee [9] reported in their study that the QOL of Korean adults with CHD was lower than that of a healthy control group, while Apers et al. reported that the overall QOL in adults with CHD was generally good in their study which covered samples from 15 countries. These inconsistent results indicate that QOL of patients with CHD varies depending on patients’ individual experiences [4].

HRQOL has been defined as “the specific impact of illness or injury, medical treatment, or health-care policies on an individual’s quality of life” [10]. Included in this concept is the individual’s perception of the impact of their disease or condition on their physical health status, psychological and social functioning, and emotional well-being, both in terms of their ability to function within these domains and their satisfaction derived from doing so [10, 11]. Accordingly, HRQOL can be affected by various factors such as health condition, peer group, and family members. Family may be a key predictor of HRQOL for adolescents with disease, because family can provides adolescents with the available information they identify as important [12].

However, only limited research has examined familial and psycho-social factors related with HRQOL for adolescents with CHD. because disease-related condition had a great impact on HRQOL for adolescents with CHD. Therefore, we explored the HRQOL of adolescents with CHD with respect to health condition and familial factors. The objective of the study was to investigate associations between health conditions and familial factors vis-a-vis the HRQOL of adolescents with CHD.

## Methods

### Design

This study was designed as a descriptive study to examine the health condition and familial factors associated with HRQOL for adolescents with CHD.

### Data collection and sample

A total of 103 adolescents aged 13–19 years undergoing follow-up at a pediatric cardiology clinic at a university-affiliated tertiary medical center in Seoul, South Korea were enrolled in this study. Between July 22 and August 23, 2013, all adolescent patients who visited the pediatric cardiology clinic for follow-up and who met the inclusion criteria were invited to participate in the study. Of these, two patients declined to complete the questionnaire due to time limitations and three patients filled out the questionnaires incompletely; all together, a total of 98 participants were included in the final analysis. Adolescents who had been diagnosed with CHD and have undergone a medical procedure or surgery were included. Adolescents who had a reading disability and insufficient communication skills were excluded. A minimum sample size of 77 was determined by using G-power analysis for F-test, multiple regression analysis with three number of predictors to achieve 80% power, an alpha = .05 and an effect size of 0.15 which is medium size [13]. A research assistant involved with this study explained our questionnaire to participating adolescents and their parents. Following the approval of participants and their families, we obtained self-reported questionnaire results from all subjects. Medical diagnosis was transcribed from the medical record.

### Measurements

Adolescents with CHD completed a self-report questionnaire, which included standardized instruments to measure their perceptions of their mother’s and father’s parental rearing behaviors, HRQOL, and socio-demographic characteristics. Additionally, participant medical records were reviewed retrospectively in order to obtain health-related factors such as diagnosis.

#### Parental rearing behaviors (FEE)

We measured perceptions of parental rearing behaviors in adolescents with CHD using the Fragebogen zum erinnerten elterlichen Erziehungsverhalten (FEE). The FEE consists of 24 items (8 in each subscale), and assesses three parenting dimensions: paternal/maternal emotional warmth, paternal/maternal rejection and punishment, and paternal/maternal control and over-protection [14]. The 24 items consist of 4-point scales, with scales in each domain ranging from eight to 32. Higher scores indicate high recognition for parental behaviors in each domain. Before using the measurement tool, the German questionnaire was translated into Korean by a bilingual expert fluent in both German and Korean. A second bilingual expert verified the meaning of each sentence using reverse translation. Seven healthy adolescents without CHD participated in a pilot test for validity before data collection from CHD participants was initiated. The internal reliability coefficients in the study for 2984 general subjects were 0.86, 0.89, and 0.74 for paternal/maternal emotional warmth, paternal/maternal rejection and punishment, and paternal/maternal control and over-protection, respectively [14]. The internal reliability coefficients in this study were 0.94, 0.87, and 0.81 for paternal/maternal emotional warmth, paternal/maternal rejection and punishment, and paternal/maternal control and over-protection, respectively.

#### Pediatric cardiac quality of life inventory (PCQLI)

We measured CHD adolescent HRQOL using the Pediatric Cardiac Quality of Life Inventory (PCQLI). The PCQLI is a disease-specific QOL measure for children and adolescents with congenital and acquired heart disease, designed to be self-administered within 10 min. It is composed of 29 items and two subscales, which are disease impacts with 17 items and psychosocial area with 12 items, measured with a five point Likert scale [15]. We used the adolescent form, in which the scores can range from zero to 100, with higher scores indicating higher QOL levels for each area. Before using the PCQLI, the researchers translated the instrument from English into Korean, and a bilingual expert verified the meaning of each sentence using reverse translation. Subsequently, five experts (including two pediatric cardiologists, one pediatric cardiac surgeon, one pediatric nurse practitioner, and one pediatric nursing professor) evaluated and modified the items in the instrument to pertain to pediatric patients with CHD. The reliability coefficient was 0.84 in the original study [15], 0.93 in a study of adolescents with heart disease [16], and 0.94 in the present study.

#### Socio-demographic characteristics and health-related characteristics

Gender, age, school, presence of siblings, having any religion, perceived economic status, and parent education levels were assigned as socio-demographic characteristics. Diagnosis and NYHA class were assigned as health-related characteristics. Diagnosis was classified as simple, moderately severe, and great complexity [17].

### Ethical considerations

Ethics approval for this human-subject database research was obtained from the institutional review board (IRB No. 2013–0597) of the hospital where this study was conducted.

### Data analysis

The data was analyzed using SPSS 21.0. Descriptive statistics were used to define participants’ socio-demographic characteristics, health-related characteristics, perceived parental rearing behaviors, and HRQOL. Student’s *t*-test and analysis of variance (ANOVA) were conducted to identify HRQOL differences according to general and health-related characteristics. Correlation analysis was used to investigate the relationship between perceived parental rearing behaviors and HRQOL. We used stepwise multiple linear regression analysis to explore the relationship among health conditions, familial factors and HRQOL for CHD adolescents. We tested independent variables including the presence of siblings, economic status, NYHA functional class, emotional warmth and control/overprotection of parents. These were significant variables associated with HRQOL in previous analyses.

## Results

### General and health-related characteristics of the participants

Participant demographics and disease-related characteristics are shown in Table 1. Among all participants, 65 (66.3%) were male with mean age of 15.18. A total of 53 participants (54.1%) were middle school students, 37 (37.8%) were high school students, and eight participants (8.2%) were university students. Most of the participating adolescents had siblings. Only six participants (6.1%) were only children. Among the participants’ parents, 65 (66.3%) fathers and 49 (50%) mothers had a university-level education. A total of 81 participants (82.7%) were self-described as having above average economic status. Disease complexity was classified in three categories [17], and 50 participants (51%) had greatly complex CHD. In addition, 67 participants (68.4%) were classified as New York Heart Association (NYHA) functional class I, and four participants (4.1%) were reported to have severe dyspnea in minimal activity (allowing for disease-related symptoms).

**Table 1:** General characteristics and health-related characteristics of the participants

Variable		*n* (%) or M ± SD
Gender	Male	65 (66.3)
	Female	33 (33.7)
Age		15.18 ± 1.86
School attendance	≤Middle	53 (54.1)
	High	37 (37.8)
	College	8 (8.2)
Presence of sibling	Yes	92 (93.9)
	No	6 (6.1)
Having any religion	Yes	45 (45.9)
	No	53 (54.1)
Living with	Parent	93(94.9)
	single	5 (5.1)
Father’s education level	<High school	4 (4.1)
	High school	29 (29.6)
	College or beyond	65 (66.3)
Mother’s education level	<High school	4 (4.1)
	High school	45 (45.9)
	College or beyond	49 (50.0)
Perceived economic status	Good	5 (5.1)
	Average	81 (82.7)
	Bad	12 (12.2)
Diagnosis	Simple	23 (23.5)
	Moderate severity	25 (25.5)
	Great complexity	50 (51.0)
NYHA	I	67 (68.4)
	II	27 (27.6)
	III	4 (4.1)

*N* = 98

### HRQOL for adolescents with CHD according to general and health-related characteristics

The mean HRQOL score (SD) was 79.50 (15.37), and ranged from 28.6 to 100. HRQOL was significantly related to the presence of siblings (80.57 vs. 63.06, *p* = 0.01), perceived economic status (87.07 vs. 80.69 vs. 68.34, *p* = 0.02), and NYHA functional class (83.42 vs. 73.71 vs. 52.93, *p* = 0.00) (Table 2).

**Table 2:** HRQOL according to general characteristics and health-related characteristics

Variable		HRQOL			
		Mean	SD	t or F	p
Gender	Male	79.33	16.77	−.15	.88
	Female	79.83	12.39		
School attendance	≤Middle	80.17	14.31	1.13	.33
	High	80.23	15.56		
	College	71.69	20.76		
Presence of sibling	Yes	80.57	14.85	−2.80	.01
	No	63.06	14.91		
Having any religion	Yes	79.24	17.69	−.15	.30
	No	79.92	13.25		
Living with	Parent	79.94	14.95	−1.24	.22
	single	71.25	22.27		
Father’s education level	<High school	77.41	28.94	.28	.76
	High school	77.94	17.04		
	College or beyond	80.33	13.77		
Mother’s education level	<High school	74.95	28.20	.36	.70
	High school	80.68	16.09		
	College or beyond	78.79	13.66		
Perceived economic status	Good	87.07	9.55	4.29	.02
	Average	80.69	13.74		
	Bad	68.34	22.47		
Diagnosis	Simple	83.25	14.88	1.39	.26
	Moderate severity	80.82	10.35		
	Great complexity	77.12	17.39		
NYHA	I	83.42	12.43	12.46	.00
	II	73.71	16.08		
	III	52.93	20.21		
Total		79.50	15.37		

*N* = 98

### Correlations between perceived parental rearing behaviors and HRQOL

Participant HRQOL was related to emotional warmth (*r* = 0.22, *p* = 0.03) and control/overprotection (*r* = −0.20, *p* = 0.04) of fathers, and emotional warmth (*r* = 0.23, *p* = 0.03) and control/overprotection (*r* = −0.22, *p* = 0.03) of mothers, which were sub-factors of parental rearing behaviors for both fathers and mothers (Table 3).

**Table 3:** Correlational relationships between perceived parenting style and HRQOL

Variables	Father			Mother		
	Emotional Warmth	Rejection/ punishment	Control/ overprotection	Emotional Warmth	Rejection/ punishment	Control/ overprotection
HRQOL	.22^*^	−.19	−.20^*^	.23^*^	−.11	−.22^*^

*N* = 98

*: *p* < 0.05

### Health condition and familial influence on HRQOL as assessed by multiple regression analysis

In order to explore health condition and familial influence on HRQOL among adolescents with CHD, stepwise multiple linear regression analysis was performed. Our results indicated that NYHA class (Adj R^2^ = .186, *p* = .000), presence of siblings (Adj R^2^ = .240, *p* = .010), and mother’s emotional warmth (Adj R^2^ = .265, *p* = .043) significantly affected CHD adolescent HRQOL and the explanatory power from the three variables was 26.5% (Table 4).

**Table 4:** Factors related to HRQOL

Variable	B	β	AdjR-sq	P
Constant	58.634			
NYHA class	−11.391	−.416	.186	.000
Presence of sibling	14.681	.230	.240	.010
Mother’s emotional warmth	0.482	.180	.265	.043

*N* = 98

F = 12.641

## Discussion

The mean score of HRQOL measured using PCQLI in this study was 79.50. In comparison with the HRQOL of adolescents with CHD in Westerns countries, the mean score of HRQOL in this study was similar to those in other studies using PCQLI. For example, the mean score (SD) of PCQLI for 781 adolescents with CHD in US was 79.7 (15.0) [16], and that for 673 adolescents with CHD from US and UK was 76.0 (16. 0) [18]. This result indicates that HRQOL of Korean adolescents with CHD was similar to that in Western countries.

This study showed that mother’s emotional warmth positively associated with the HRQOL for CHD adolescents. Consistent with this study, results from a study by Rassart et al. [19] indicated that perceptions of parental acceptance were positively correlated with CHD adolescent self-esteem. Warm and accepting parent-adolescent relationships contributed to the well-being of children and adolescents at risk [20], and were related to higher levels of QOL and fewer psychological symptoms in adolescents with chronic diseases [21]. Also, The QOL of adolescents suffering from inflammatory bowel disease positively correlated with the warm parental style [22] while the QOL of adolescents with diabetes type 1 was positively associated with mindful parental style [23]. Our findings indicate that emotional warmth is positively associated with the HRQOL of adolescents with CHD. Therefore, health providers need to inform parents of the advantages of demonstrating these positive parental behaviors.

This study also found the presence of siblings was positively associated with the HRQOL of adolescents with CHD. This finding is consistent with the study for the siblings of people diagnosed with a mental disorder that showed siblings mature faster and support their family [24]. Siblings played a significant role as emotional support and advocates [25] thus, siblings could facilitate adaptation and enhance the resilience of adolescents with disease [25]. The presence of siblings may be vital in helping to maintain a sense of security and emotional continuity, as well as self-identity [26]. Therefore, further studies on the development of programs that include siblings as well as parents for adolescents with CHD are needed.

According to this study’s results, the HRQOL for adolescents with CHD did not differ according to CHD severity. That is, disease severity was a poor HRQOL predictor [27, 28]. However, the HRQOL for adolescents with CHD in this study was significantly different depending on their NYHA functional class. Consistent with this finding, Schoormans et al. [29] used a TNO/AZL Adult Quality of Life-CHD (TAAQOL-CHD) subscale and showed that perceived HRQOL in CHD patients was partially associated with their NYHA functional class. In contrast to this finding, a study by Bang et al. [30] suggested that QOL levels, depression, and anxiety in CHD patients did not differ significantly according to their NYHA class. The NYHA class for participants in that study, however, consisted of only classes I and II. Accordingly, the effects on QOL by NYHA class need to include patients in classes I, II, and III for more robust results. Also, Bang et al. [30] used the short version of the World Health Organization QOL (WHOQOL-BREF) to evaluate QOL. Evaluation of HRQOL using WHOQOL-BREF is difficult because WHOQOL is generic measures of QOL, and may not capture the nuances of QOL with a chronic disease. Therefore, future research is needed to assess QOL using a scale for CHD-related HRQOL.

The explanatory power from NYHA class, presence of siblings, and mother’s emotional warmth in this study was 26.5%, which showed the low level of explanation. This indicates that other variables besides our results might affect to HRQOL of adolescents with CHD. Researchers mentioned that illness perception [31], emotional distress [32] and social support [32] were also able to be factors that influenced HRQOL of adolescents with CHD. In addition, gender, school and perceived economic status were excluded as related factors on HRQOL of adolescents with CHD in this study. Aper et al. [4] insisted that gender was not associated with HRQOL in their study for QOL of adults with CHD, whereas other researchers mentioned that gender was an influential factor on HRQOL in the studies for adult patients with heart disease [31, 32]. With regard to economic statue, Eslami et al. [32] described that economic status was associated with HRQOL of adults with CHD, which is not inconsistent with our study. However, the majority of studies for examining the relation between QOL and socio-demographic variables were for adults with CHD. Therefore, future research is needed to investigate influential factors of HRQOL in adolescent with CHD including illness perception, emotional distress, peer-support, and so on.

### Limitations

This study has several limitations. First, participants came from a single medical center in Korea and parental rearing behaviors of Korean cannot represent all parents, possibly limiting the generalizability of these findings. Second, there is potential bias from the translation of the scales, such as FEE and PCQLI, to Korean without consideration of cultural differences. Third, data collecting period was only summer time which can affect HRQOL, possibly limiting the representativeness. Forth, it is difficult to describe the factors affecting the HRQOL of adolescents with CHD, because this study was designed as a cross-sectional study; therefore, follow-up research designed by a longitudinal study is needed. Lastly, the number of participants without a sibling was six and the number of participants with NYHA class III was four, which are small sample sizes; therefore, a larger follow-up study on the presence of siblings and NYHA class III participants is needed.

## Conclusions

Our results indicate that NYHA class, presence of siblings, and mothers’ emotional warmth are predictors of HRQOL for adolescents with CHD. To improve the CHD adolescent HRQOL, emotionally warm parental rearing behaviors, the presence of siblings, and lower level of NYHA class are important familial factors. Therefore, it is important for healthcare providers to develop greater sensitivity to familial factors as well as the exigencies of the CHD health condition.

## Abbreviations

CHD: Congenital heart disease
FEE: Parental rearing behaviors
HRQOL: Health-related quality of life
NYHA: NYHA New York Heart Association
PCQLI: Pediatric cardiac quality of life inventory
TAAQOL-CHD: TNO/AZL Adult Quality of Life-CHD
WHOQOL-BREF: World Health Organization QOL short version
